# Diagnosis and management of Parkinson’s disease dementia and dementia with Lewy bodies: recommendations of the Scientific Department of Cognitive Neurology and Aging of the Brazilian Academy of Neurology

**DOI:** 10.1590/1980-5764-DN-2022-S105PT

**Published:** 2022-11-28

**Authors:** Jacy Bezerra Parmera, Vitor Tumas, Henrique Ballalai Ferraz, Mariana Spitz, Maira Tonidandel Barbosa, Jerusa Smid, Breno José Alencar Pires Barbosa, Lucas Porcello Schilling, Márcio Luiz Figueiredo Balthazar, Leonardo Cruz de Souza, Francisco Assis Carvalho Vale, Paulo Caramelli, Paulo Henrique Ferreira Bertolucci, Márcia Lorena Fagundes Chaves, Sonia Maria Dozzi Brucki, Ricardo Nitrini, Raphael Machado Castilhos, Norberto Anízio Ferreira Frota

**Affiliations:** 1 Universidade de São Paulo, Faculdade de Medicina, Departamento de Neurologia, Grupo de Neurologia Cognitiva e do Comportamento, São Paulo SP, Brasil.; 2 Universidade de São Paulo, Faculdade de Medicina de Ribeirão Preto, Departamento de Neurociências e Ciências do Comportamento, São Paulo SP, Brasil.; 3 Universidade Federal de São Paulo, Escola Paulista de Medicina, Departamento de Neurologia e Neurocirurgia, São Paulo SP, Brasil.; 4 Universidade do Estado do Rio de Janeiro, Serviço de Neurologia, Rio de Janeiro RJ, Brasil.; 5 Universidade Federal de Minas Gerais, Faculdade de Medicina, Departamento de Medicina Interna, Belo Horizonte MG, Brasil.; 6 Faculdade Ciências Médicas de Minas Gerais, Medicina Geriátrica, Belo Horizonte MG, Brasil.; 7 Universidade Federal de Pernambuco, Centro de Ciências Médicas, Área Acadêmica de Neuropsiquiatria, Recife PE, Brasil.; 8 Instituto de Medicina Integral Prof. Fernando Figueira, Recife PE, Brasil.; 9 Pontifícia Universidade do Rio Grande do Sul, Escola de Medicina, Serviço de Neurologia, Porto Alegre RS, Brasil.; 10 Pontifícia Universidade do Rio Grande do Sul, Instituto do Cérebro do Rio Grande do Sul, Porto Alegre RS, Brasil.; 11 Pontifícia Universidade do Rio Grande do Sul, Programa de Pós-Graduação em Gerontologia Biomédica, Porto Alegre RS, Brasil.; 12 Universidade Estadual de Campinas, Faculdade de Ciências Médicas, Departamento de Neurologia, Campinas SP, Brasil.; 13 Universidade Federal de Minas Gerais, Departamento de Clínica Médica, Belo Horizonte MG, Brasil.; 14 Universidade Federal de São Carlos, Centro de Ciências Biológicas e da Saúde, Departamento de Medicina, São Carlos SP, Brasil.; 15 Hospital de Clínicas de Porto Alegre, Serviço de Neurologia, Porto Alegre RS, Brasil.; 16 Universidade Federal do Rio Grande do Sul, Faculdade de Medicina, Serviço de Neurologia, Porto Alegre RS, Brasil.; 17 Hospital Geral de Fortaleza, Serviço de Neurologia, Fortaleza CE, Brasil.; 18 Universidade de Fortaleza, Fortaleza CE, Brasil.

**Keywords:** Consensus, Parkinson Disease, Lewy Bodies, Dementia, Consenso, Doença de Parkinson, Corpos de Lewy, Demência

## Abstract

Parkinson’s disease dementia (PDD) and dementia with Lewy bodies (DLB) represent the second most common type of degenerative dementia in patients aged 65 years and older, leading to progressive cognitive dysfunction and impaired quality of life. This study aims to provide a consensus based on a systematic Brazilian literature review and a comprehensive international review concerning PDD and DLB. Moreover, we sought to report on and give recommendations about the best diagnostic approaches focusing on primary and secondary care. Based on the available data, we recommend clinicians to apply at least one brief global cognitive instrument to assess PDD, such as the Mini-Mental State Examination and preferably the Montreal Cognitive Assessment and the Addenbrooke’s Cognitive Examination-Revised. Validated instruments to accurately assess functional abilities in Brazilian PD patients are still incipient. Further studies should focus on biomarkers with Brazilian cohorts.

## RESUMO

A demência da doença de Parkinson (DDP) e a demência com corpos de Lewy (DCL) representam a segunda causa mais comum de demência neurodegenerativa em pessoas com mais de 65 anos, ocasionando progressivo declínio cognitivo e comprometimento da qualidade de vida. O presente estudo tem como objetivo prover um consenso de especialistas sobre a DDP e DCL, baseado em revisão sistemática da literatura brasileira e revisão não-sistemática de literatura internacional. Ademais, tal estudo visa promover informação e conceder recomendações sobre abordagem diagnóstica, com foco nos níveis de atenção primária e secundária em saúde. Com base nos dados disponíveis, recomendamos que os profissionais realizem pelo menos um breve instrumento cognitivo global, como o Mini-Exame do Estado Mental, contudo de preferência optem pela Avaliação Cognitiva de Montreal e o Exame Cognitivo de Addenbrooke-Revisado. Observa-se uma carência de instrumentos validados para a avaliação precisa das habilidades funcionais em pacientes brasileiros com DDP e DCL. Além disso, mais estudos focando em biomarcadores com coortes brasileiras também são necessários.

## INTRODUCTION

Parkinson’s disease dementia (PDD) and dementia with Lewy bodies (DLB) are different clinical syndromes that share the same pathological hallmark, namely Lewy body disease, in which post-mortem examination shows neuronal α-synuclein inclusions (Lewy bodies) and neuronal loss. The umbrella term Lewy Body Dementia (LBD) includes both of these syndromes, representing the second most common type of degenerative dementia in patients aged 65 years and older and leading to progressive cognitive dysfunction, motor deterioration, and impaired quality of life^1^ ^),(^ ^2^.

Despite having the same pathological substrate, Parkinson’s disease (PD) and DLB are classified as different diseases based on the temporal relationship of cognitive and motor symptoms. Current criteria recommend diagnosing a patient with PDD when dementia develops in the context of well-established PD^1^ and with DLB when dementia precedes or coincides within one year of the development of motor symptoms, namely the “1-year rule”. This is considered an empirical approach that avoids clinical practice mistakes and clarifies the distinction in research and clinical studies^2^.

PDD and DLB significantly affect one’s psychological and social life, decreasing the quality of life for both patients and caregivers. In Brazil, most PD patients are assisted in their homes by family members, who act as informal caregivers. Caregivers of PD patients face increasing burdens and may develop burnout, depression, and anxiety. The most significant predictors of burden in Brazilian PD caregivers are cognitive and behavioral symptoms in PD patients, the time of caregiving, and the occurrence of mood disorders in caregivers^3^ ^),(^ ^4^.

This work aimed to reach a specialist consensus based on a systematic Brazilian literature review and a comprehensive international review of other updated and relevant literature on PDD and DLB. Moreover, we sought to report on and provide a clinical guide with recommendations focusing on primary and secondary care in the workup of PDD and DLB.

## METHODS

This study was conceptualized in meetings held from April to June 2021 to design the review process and draft the consensus. The consensus group comprised seven members experts in the field.

Firstly, we performed a systematic review of the Brazilian literature concerning epidemiological, clinical, ancillary tests, and biomarkers and of management studies regarding PDD and DLB. A systematic computer-based literature search using the Start program was performed on the PubMed, Scielo, and PsycINFO electronic databases. For the search, the medical subject headings [“Parkinson’s disease”] OR [“Lewy Body dementia”] OR [“diffuse Lewy Body disease”] AND [“dementia”] OR [“mild cognitive impairment”] OR [“cognitive dysfunction”] and (Brazil) using English and Portuguese and human studies filters. Inclusion criteria were 1) Brazilian studies referring to PD and DLB 2) investigating cognitive or associated features 3) in older adults or those over 18 years old. Exclusion criteria were 1) studies focusing on other cognitive primary diagnoses.

The consensus group was later divided into three subgroups concerning epidemiology and risk factors, clinical features and neuropsychological assessment, or biomarkers and management, each subgroup in charge of critically reviewing the literature selected based on predefined selection criteria. A comprehensive literature search was also performed to add current information and knowledge for insufficient data acquired from the systematic review or if the specialists wanted to enrich the consensus study.

## RESULTS

**Figure 1:**
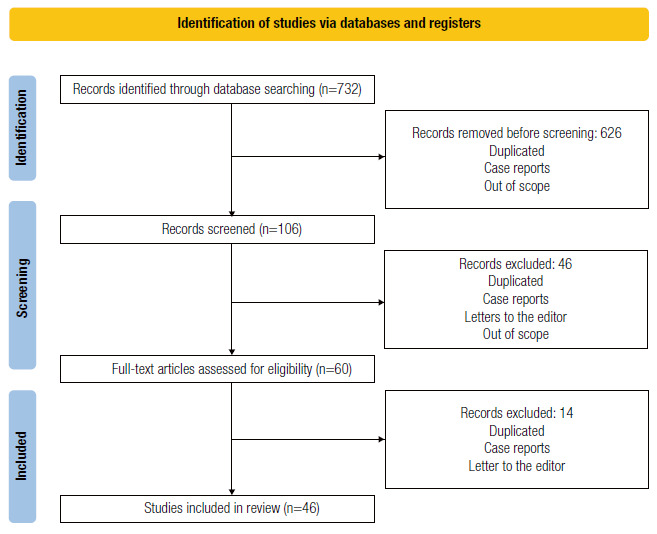
Prisma flow diagram.

The search gathered 732 records identified by database searching. After an initial exclusion, 106 articles published in Brazilian cohorts were selected, of which 60 were later excluded due to the reasons explained in Figure 1.

In total, 46 articles were included in this consensus. For topics without specific articles in the Brazilian literature, 17 studies, including research articles, review articles, and international consensus, were added to the 46 previously selected Brazilian articles, in a sum of 63 studies.

The PRISMA flow diagram summarizes the search (Figure 1).

### Epidemiology

The prevalence rate of parkinsonism in two community studies with older adults aged 60 years and over in Brazil ranged from 7.2%^5^ to 10.6%^6^, with idiopathic Parkinson’s disease being the most frequent etiology. Both studies observed a progressive increase of parkinsonism with age, reaching 30.4% prevalence in the population over 95 years old^6^. Around 15% of patients with parkinsonism had dementia in the Bambuí study^5^ ^)^ and 56.7% had it in the Pietá study^6^. In community-based studies, PDD represented 13.5% of cases with parkinsonism and dementia^6^. DLB, in turn, caused less than 5% of cases^7^ ^),(^ ^8^.

In tertiary centers-based studies, the frequency of these two etiologies ranged from 3.7% to 15% of cases^9^ ^)-(^ ^13^ Similarly, a study based on neuropathological diagnosis cases^14^ showed that LBD caused 15% of cases of dementia, whether isolated or associated with other pathologies. LBD was also the second most frequent neurodegenerative etiology of dementia and related to a higher risk of dementia with an odds ratio (OR) of 3.4 (CI: 1.94-5.97). The same study had high specificity for clinical diagnosis^14^.

Dementia frequently occurs in PD and its prevalence increases with disease duration, ranging from 23% in the first years of the disease to 80% after over 15 years of symptoms^15^ ^)-(^ ^17^. Understanding which risk factors are associated with the occurrence of dementia in PD patients and what differentiates LBD patients from other etiologies is essential for an earlier diagnosis and more appropriate therapy for the disease. These aspects will be discussed in the following topics.

### Recommendations for the diagnosis of PDD and DLB

Dementia is usually defined by clinical diagnostic criteria. Historically, the most used criteria, even for patients with PD, were those of the various versions of the Diagnostic and Statistical Manual of Mental Disorders (DSM). This concept changed in 2007 when a task force of the Movement Disorder Society (MDS) developed specific diagnostic criteria for PDD^1^ and recommended procedures to operationalize the diagnosis^18^. Since then, clinical studies, including Brazilian studies, have used these criteria and procedures the most. We recommend using these criteria with a few adaptations marked below with quotation marks (Table 1).

**Table 1:** Criteria for the diagnosis of probable and possible Parkinson’s disease dementia^1^.

***The diagnosis is probable when:***
**A.** Core features must be both present.
1. Diagnosis of Parkinson’s disease according to specific “diagnostic criteria” *
2. A syndrome of “cognitive decline” with insidious onset and slow progression, developing within the context of established PD and diagnosed by history, clinical, and mental examination, defined as:
- Impairment in more than one cognitive domain (including: attention, executive functions, visuo-spatial functions, memory and language)
- Decline from a premorbid level of functioning
- Deficits severe enough to impair daily life (social, occupational, or personal care), regardless of the impairment from motor or autonomic symptoms
**B.** Associated clinical features.
Typical profile of cognitive deficits including impairment in at least two of the four core cognitive domains (impaired attention which may fluctuate, impaired executive functions, impairment in visuo-spatial functions, and impaired free recall which usually improves with cueing)
Behavioral features such as apathy, changes in personality and mood, hallucinations, delusions, and excessive daytime sleepiness may be present (but are not necessary for diagnosis)
**C.** Features which do not exclude PDD, but make the diagnosis uncertain.
Existence of any other abnormality which may cause cognitive impairment but is not as the cause of dementia, e.g., presence of relevant vascular disease in imaging.
The time interval between the development of motor and cognitive symptoms is unknown**
**D.** There are none of the following features suggesting other conditions or diseases as the cause of mental impairment, which would hinder an accurate diagnosis of PDD: delirium, diagnosis of major depression, evidence for diagnosis of probable vascular dementia.
***The diagnosis is possible when:***
**A.** Core features must be both present.
**B.** The cognitive decline presents with atypical profile of cognitive impairment in one or more domains, such as prominent or receptive (fluent) aphasia or pure storage-failure type amnesia (memory does not improve with cueing or in recognition tasks) with preserved attention.
*or*
**C.** There are features that make the diagnosis uncertain (e.g., presence of relevant vascular disease in imaging).
*or*
**D.** Time interval between the development of motor and cognitive symptoms is unknown (“*1-year rule*”)
*or*
**E.** There are features suggesting other conditions or diseases as causes of mental impairment (delirium, diagnosis of major depression, evidence for diagnosis of probable vascular dementia)

* United Kingdom Parkinson’s Disease Society Brain Bank diagnostic criteria (20) for PD or another validated criterion; ** Refers to the “1-year rule” to differentiate PDD from Lewy body dementia. PDD develops within the context of established PD or arbitrarily at least a year after the onset of the classic motor symptoms.

The fourth consensus report of the DLB Consortium, in turn, has recently refined the recommendations for the clinical and pathologic diagnosis of DLB^19^. It incorporated recent developments to increase sensitivity and clearly distinguished between clinical features and diagnostic biomarkers. The report classified clinical symptoms and signs as core or supportive and weighed biomarkers as indicative or supportive based on their specificity and the volume of high-quality evidence available. We also recommend the use of these criteria in clinical practice (Table 2).

**Table 2:** Criteria for the diagnosis of probable and possible dementia with Lewy bodies^2^.

1. Essential: dementia is required for a diagnosis of DLB and defined as a progressive cognitive decline which affects social and occupational functions or daily activities. It mainly affects attention, executive function, and visuoperceptual abilities, worsening memory impairment as it progresses.
Core clinical features:
Fluctuating cognition with pronounced variations in alertness.
Recurrent visual hallucinations.
REM sleep behavior disorder.
One or more spontaneous cardinal features of parkinsonism (bradykinesia, rest tremor, or rigidity).*
Supportive clinical features: severe sensitivity to antipsychotic agents, postural instability, repeated falls; syncope or other transient episodes of unresponsiveness; severe autonomic dysfunction, e.g., constipation, orthostatic hypotension, urinary incontinence; hypersomnia; hyposmia; other hallucination modalities; systematized delusions; apathy, anxiety, and depression.
Indicative biomarkers:
Reduced dopamine transporter uptake in basal ganglia demonstrated by SPECT or PET.
Abnormal (low uptake) 123iodine-MIBG myocardial scintigraphy.
Polysomnographic confirmation of REM sleep without atonia.
Supportive biomarkers:
Relative preservation of medial temporal lobe structures on CT/MRI scan.
Generalized low uptake on SPECT/PET perfusion/metabolism scan with reduced occipital activity. Cingulate island sign on FDG-PET imaging.
Prominent posterior slow-wave activity on EEG with periodic fluctuations in the pre-alpha/theta range.
***Diagnosis is probable when:***
Two or more core clinical features are present
OR
Only one core clinical feature is present but with one or more indicative biomarkers.
***Diagnosis is possible when:***
Only one core clinical feature (with no biomarkers)
One or more indicative biomarkers are present without core clinical features.

*We continue to recommend the existing 1-year rule between the onset of dementia and parkinsonism.

We suggest that the diagnosis of PD should use validated diagnostic criteria, which can be either the United Kingdom Parkinson’s Disease Society Brain Bank diagnostic criteria^20^ or any other diagnostic criteria with established accuracy for diagnosis, such as the recently proposed MDS Parkinson’s Disease criteria^21^.

We also suggest maintaining the “1-year rule” for clinical and research purposes, despite the controversy over whether PDD and DLB are the clinical spectra of the same disease. In turn, specialists from the MDS have recently proposed not using the “1-year rule” to distinguish PDD from DLB^21^. Nevertheless, this approach has been criticized and does not present general agreement.

We recommend adopting the formal criteria for PD-mild cognitive impairment (MCI) published in 2012^22^ to assess the progression of cognitive decline in PD patients. Proposed research criteria for diagnosing prodromal DLB have recently introduced three possible presentations to the prodromal phase: MCI, delirium-onset, and psychiatric-onset. We recommend adopting the DLB-MCI criteria^19^.

### Clinical features in PDD and DLB

The evaluations of PDD and DLB present two major challenges. Regarding PDD, it is the prediction and early identification of the progression of cognitive decline to dementia. In DLB, it is probably the differentiation from Alzheimer’s disease (AD) ^(^ ^23^.

Parkinson’s Disease is mainly characterized by classic motor symptoms, such as resting tremor, rigidity, and bradykinesia. However, it is also associated with many non-motor manifestations that are well-recognized symptoms and predominant at the late stages of the disease, such as hyposmia, constipation, depression, and rapid eye movement (REM) sleep behavior disorder (RBD). These may precede the motor signs whereas dementia and psychosis are common in the late stages of the illness^1^ ^),(^ ^20^. DLB has the same non-motor symptoms, but the cognitive decline occurs earlier and the motor symptoms are milder, sometimes absent. Dementia is essential to diagnose an individual with DLB.

PDD and DLB cognitive profiles are characterized by impairment mainly in attention, executive, and visuospatial functions. Behavioral symptoms such as affective changes, hallucinations, and apathy are also frequent^1^. Most studies with Brazilian samples showed the same pattern of cognitive impairment^24^ ^),(^ ^25^. On the other hand, episodic memory is less affected and patients with MCI usually have more difficulty in free recall than cued recall in memory tests (such as a list of words or pictures). A previous study exploring visuospatial dysfunctions in PD patients showed that 62.2% of 35 patients could not copy the pentagon drawing^26^. Moreover, Machado et al. suggested that language and visual organization tend to follow motor skills and general cognitive performance in patients with DLB^27^. Poor cognitive performance frequently correlates with more advanced stages of the disease, older age, low schooling level, depression, and poorer quality of life^15^ ^),(^ ^17^ ^),(^ ^28^. Cardiovascular risk factors and PDD, however, are not correlated with each other^29^.

The main cognitive feature in DLB is fluctuating cognition, which represents a fluctuation in attention or level of consciousness. This feature can range from episodes of inattention and mental confusion, with disorganized thinking and behavior, to lethargy and excessive daytime sleepiness. DLB and PD have differences of parkinsonism presentation. DLB presents more symmetrical parkinsonism, less frequent rest tremor, and greater postural instability and lower responsiveness to levodopa^2^.

REM Behavior Disorder (RBD) has a high specificity for suggesting an alpha-synucleinopathy. RBD is a parasomnia characterized by loss of atony during REM sleep. The current DLB^2^ criteria included this feature among the core clinical manifestations of the disease. Clinical suspicion is based on the patient history, in which the caregiver usually describes that the patient has abnormal vocalizations, aberrant motor behavior, and vivid dreams. The suspicion is usually confirmed by polysomnography (PSG), but questionnaires can be used clinically if PSG is unavailable. The Brazilian Portuguese version of the RBD screening questionnaire (RBDSQ) for patients with PD may be helpful for RBD diagnosis in the country^28^. This disorder should be actively questioned since an individual could develop it up to 15 years before developing dementia or parkinsonism. Diagnosing this disorder is therefore important to improve patient’s quality of life.

Hallucinations are one of the few features that usefully distinguish between DLB/PDD and AD. The phenomenology of hallucinations in PDD and DLB is very similar. Visual hallucinations occur twice as frequently as auditory ones, being mostly complex, formed hallucinations of anonymous people, but they may also be family members, body parts, animals, or machines^1^ ^),(^ ^30^. Overall, patients with PDD seem to have less frequent or less severe psychiatric symptoms than patients with DLB. Such differences, however, may simply reflect the disparity in the overall dementia severity between PDD and DLB^1^ ^),(^ ^31^.

Depression, another non-motor symptom frequent in PD patients, can worsen executive dysfunction, especially in subjects with low schooling level^32^. Other supportive clinical features in DLB are: severe sensitivity to antipsychotic agents, postural instability, repeated falls, syncope, or other transient episodes of unresponsiveness; severe autonomic dysfunction, hypersomnia, hyposmia, other hallucination modalities; systematized delusions; and apathy, anxiety, and depression^2^.

### Which diagnostic procedures and neuropsychological tests should be used to diagnose and evaluate PDD and DLB?

Many different methods and instruments can be used to assess cognition in patients with PD and DLB.

We recommend using an adapted version of the procedures to operationalize the diagnostic criteria proposed by the MDS for PDD diagnosis ^(^ ^18^. These criteria suggested two levels of diagnosis for PDD. In level I, the diagnosis is based upon using straightforward procedures and bedside cognitive tests, which a non-specialist would easily apply in clinical consultations. In level II, diagnosis would require a more extensive and specialized neuropsychological evaluation.

Predictably, a previous Brazilian study showed that the prevalence of PDD diagnosis was higher when the more elaborate procedures of level II rather than of level I were used (23.8% versus 14.9%, respectively). This indicates that level I procedures had lower sensitivity but greater specificity than level II for the diagnosis of dementia^15^.

The MDS proposed practical procedures for level I diagnosis of PDD based on an algorithm or checklist. Both of these procedures are advantageous, either for clinical routine or clinical research. However, they require some adaptations that we suggest in Table 3.

**Table 3:** Modified algorithm for diagnosing Parkinson’s disease dementia at Level I.

Diagnosis of Parkinson’s disease*
Motor symptoms of PD developed at least “1-year” before the onset of dementia
Cognitive deficits severe enough to impact daily living**
Abnormal performance on a brief global cognitive scale***
Cognitive abnormalities affect typical cognitive domains such as: attention, executive functions, visuo-spatial functions, memory and language (excluding prominent aphasia)
Absence of Major Depression
Absence of delirium
There are no other features that make the diagnosis uncertain or suggest other conditions or diseases as causes of mental impairment (diagnosis of probable vascular dementia)
If all items are checked, the diagnosis is probable PDD
If item 2 and/or item 5, and/or item 6, and/or item 7, and/or item or 8 are not checked, the diagnosis is possible PDD

*Based on specific diagnostic criteria; **According to any method of functional assessment; ***Based on population normative levels, with scores adjusted for schooling level and other factors (age, sex, etc.) if necessary.

Regarding the assessment of functionality, the best functional assessment tool for impairment from cognitive loss in PD patients is still undefined. Most studies used functional assessment instruments applied in other dementias, and no proper validation instrument is available for patients with PD. The MDS primary recommendation proposed the “pill questionnaire” as a simple tool to define independence loss. According to this instrument, patients without functional loss are those who can describe in detail their drug schedule treatment^18^. However, many studies suggested that the questionnaire was insufficiently accurate. Accordingly, two Brazilian studies suggested that the pill questionnaire was less sensitive than other instruments to detect functional impairment^15^ ^),(^ ^33^.

Studies with Brazilian PD patients have used several instruments to assess functional abilities, including the Pfeffer Functional Activities Questionnaire (FAQ), the Disability Assessment for Dementia (DAD), the Informant Questionnaire on Cognitive Decline in the Elderly (IQCODE), Pill questionnaire, the phone call test, and the Direct Assessment of Functional Abilities (DAFA) ^(^ ^15^ ^),(^ ^33^ ^)-(^ ^37^. Two studies evaluated the PFAQ accuracy to detect functional impairment in patients with PD^33^ ^),(^ ^37^. Oliveira et al. proposed that a score >2 had 23% sensitivity and 74% specificity to predict abnormal performance on a global cognitive test^33^. In turn, Almeida et al. defined a score >3 as the best cutoff for a modified version of the PFAQ (mPFAQ) to diagnose PDD (47% sensitivity and 88% specificity). In this modified version, the authors suppressed items 5 (make coffee) and 6 (prepare a meal) from the original version to minimize the possible effects of motor symptoms that affect the interpretation of the patient’s functional assessment^37^. The authors used the IQCODE as a reference to diagnose functional impairment with the cutoff score of 3.27, the same defined for the cross-cultural adaptation of the scale for Brazilian patients with Alzheimer’s disease dementia^37^.

Baldivia et al. used DAD to assess functional impairment in patients with PD with the cutoff score of 94.6, the same previously defined for diagnosis of Alzheimer’s disease dementia in Brazilian patients^15^. In turn, Breder et al. used a modified version of DAD to evaluate patients with PD. The authors did not evaluate a disability but the dependence in activities of daily living (ADL). They classified the patient as dependent if he needed assistance for over half of the time on at least one activity evaluated due to cognitive impairment^35^. Oliveira et al. showed that close informants usually overestimate patients’ instrumental abilities in ADL. In some patients, therefore, the assessment of functional loss might only be considered reliable with direct assessment tools. The direct assessment of functional abilities might better predict impairment on a global cognitive scale than questionnaires^33^.

These studies show a lack of validated instruments to accurately assess functional abilities in Brazilian PD patients. Further studies should focus on developing these instruments. We recommend that despite the functional assessment tool used, the examiner should always be sure that other PD symptoms, such as motor symptoms, are not interfering with the patient’s ability to carry out activities. For clinical practice, though the pill questionnaire seems to be an interesting procedure for screening, it should be complemented by other functional assessments or a clinical interview aimed at functional status. For clinical research studies, authors must precisely define the methods used to assess the functional abilities of patients with PD.

Regarding global cognitive assessment, we suggest using other global cognitive scales besides the Mini-Mental State Examination (MMSE). The MDS has proposed the MMSE as a first-line assessment tool for global cognitive efficiency in PD because of the instrument’s simplicity and wide use in dementia. However, other studies, including some with Brazilian populations, showed that the MMSE had lower sensitivity than other scales to detect cognitive abnormalities in PD patients^34^ ^),(^ ^35^ ^),(^ ^38^. Moreover, MMSE cutoff scores adjusted for schooling level from normative studies performed in the Brazilian population were more accurate for diagnosis of PDD^15^ ^),(^ ^34^. Other cognitive scales should also consider schooling level.

Diagnostic accuracy also depends on the quality of evidence for normative data of cognitive scales in the Brazilian population. Therefore, a less precise approach to define cutoff scores for PDD diagnosis relies on scale-assessment studies to distinguish between PD without dementia and PDD. Souza et al. proposed using the interlocking finger test (ILFT) to screen PD patients for dementia^39^. ILFT is a simple test in which the patient is asked to imitate four meaningless bimanual gestures. The authors showed that a score <3 (each gesture earned one point) presented 61% sensitivity and 85% specificity to diagnose PDD, indicating ILFT as a practical bedside test to assess cognitive impairment in patients with PD^39^.

The Montreal Cognitive Assessment (MoCA) and the Addenbrooke’s Cognitive Examination-Revised (ACE-R) are other brief global cognitive instruments that have been used in PD and DLB. They have already been translated and validated for Brazilian populations^40^ ^)-(^ ^42^. Some studies suggest that these instruments may be more effective than the MMSE to detect dementia in PD patients^35^ ^),(^ ^36^ ^),(^ ^38^ ^),(^ ^43^ ^),(^ ^44^. However, most studies showed inadequate accuracy to evaluate PD-MCI^43^. A previous study evaluating the MoCA subtests showed that PD patients with low schooling level might have difficulty completing the tests, contributing to poor diagnostic accuracy and affecting the detection of MCI^45^. Table 4 summarizes the findings and cutoff scores applied with brief global cognitive instruments in Brazilian PD patients.

**Table 4:** Clinical Brazilian research studies with brief global cognitive instruments in Parkinson’s disease dementia.

Study	Sample characteristics	Diagnosis of dementia	Results
Sobreira et al. 2015^43^	79 PD patients	According to level II of the MDS diagnostic criteria	The area under the ROC curve for the MoCA dementia diagnosis was 0.86 (95%CI= 0.76-0.95). The best cutoff score for MoCA to differentiate patients with PDD from the others was <21 (sensitivity of 94%, specificity of 68%).
	Median age 63 years (28-81)		The area under the ROC curve for the ACE-R was 0.84 (95%CI= 0.74-0.94). The best cutoff score for ACE-R to differentiate patients with PDD from the others was **<76** (sensitivity of 88%, specificity of 68%).
	Median schooling time 6.5 years (1-20)		
	36% male		
Rocha et al. 2014^38^	70 PD patients	According to level II of the MDS diagnostic criteria	The area under the ROC curve for the MMSE was 0.88 (95% CI: 0.78-0.97). The best cutoff score for MMSE to differentiate patients with PDD from the others was **≤24**, (sensitivity of 78.5%, specificity of 96.4%).
	Mean age 64.1 years (SD= 9.3)		The area under the ROC curve for the ACE-R was 0.93 (95% CI: 0.86-0.98). The best cutoff score for ACE-R to differentiate patients with PDD from the others was **≤72** (sensitivity of 89.3%, specificity of 84.6%)
	Mean schooling time 5.9 years (SD= 3.4)		
	57.1% male		
Souza et al, 2016^39^	101 PD patients	According to MDS criteria but did say if level I or II	The area under the ROC curve for the ILFT was 0.761The best cutoff score for ILFT to differentiate patients with PDD from the others was <3 (sensitivity of 61%, specificity of 85%).
	Mean age 62.5 years (SD= 12.1)		The area under the ROC curve for the MMSE was 0.841. The best cutoff score for MMSE to differentiate patients with PDD from the others was <25 (sensitivity of 80.5%, specificity of 73%).
	Mean schooling time 5.2 years (SD= 4.1)		
	42.5% male		
Almeida et al, 2019^36^	89 PD patients	According to level II of the MDS diagnostic criteria	The best cutoff score for MoCA to differentiate patients with PDD from those with PD-MCI was <18 (sensitivity of 85.5%, specificity of 81.6%).
	Mean age 59 years		
	Mean education 8.4 years		
	53.9% male		
Camargo et al, 2016^44^	50 PD patients	According to MDS criteria but did not indicate if level I or II	The MoCA test showed an AUC=0.906 area under the ROC curve with a cutoff score of ≤19 points (sensitivity of 87.80%, specificity of 88.89%). The MMSE had an 0.936 area under the curve with a cutoff score of ≤26 points (specificity of 66.67%, sensitivity of up to 90.24%).
	Mean age 69.28 (SD= 11.41)		
	Mean schooling time 6.9 years		
	64% male		

PD: Parkinson’s disease; PDD: Parkinson’s disease dementia; MDS: Movement Disorders Society; ILFT: interlocking finger test.

Studies conducted in Brazil have assessed the cognitive profile of patients with PD using comprehensive and formal neuropsychological tests, such as the Mattis Dementia Rating Scale (MDRS), Scales for Outcomes in Parkinson’s Disease-Cognition (SCOPA-COG), Wisconsin Card Sorting Test (WCST), Frontal Assessment Battery (FAB), Verbal Fluency Tests^27^ ^),(^ ^46^, and Trail Making tests^47^. A study applying a comprehensive neuropsychological battery showed cognitive impairment in 56.7% of PD patients in a waiting list for deep brain stimulation (DBS) implantation according to FAB scores and in 76.7% of them according to MoCA, showing the importance of a formal cognitive evaluation in this group of candidates to DBS^48^. Moreover, another study showed that the Consortium to Establish a Registry for Alzheimer’s Disease (CERAD) neuropsychological battery could effectively assess cognitive deficits in PD patients^49^.

Based on the available data, we recommend clinicians to apply at least one brief cognitive instrument, such as MMSE and preferably the MoCA or ACE-R, to investigate PDD and DLB - especially since we found no Brazilian studies or samples concerning neuropsychological profiles of the latter. However, for a tertiary center and other treatment strategies such as DBS, a comprehensive neuropsychological evaluation is more appropriate if suitable.

### Ancillary investigation and biomarkers

Besides analyzing the clinical history and conducting neurological examination with brief cognitive instruments, we also recommend applying ancillary tests to confirm the PDD and DLB diagnoses. Routine screening tests are recommended to exclude other causes of cognitive impairment. We suggest conducting a complete blood count, as suggested in article one, and using structural imaging such as magnetic resonance imaging (MRI) or computed tomography (CT) scans to exclude other causes for dementia syndromes, such as vascular dementia, stroke, and brain tumors.

The PDD and DLB biomarkers do not define the presence of alpha-synuclein in vivo, but seek to reflect the repercussions of the pathophysiological process. Specifically for PDD, the current diagnostic criteria do not recommend structural, functional, or electrophysiological studies for routine diagnostic purposes in differentiating PD from PDD due to the lack of specificity^1^. Nevertheless, the current MDS criteria for PD diagnosis^21^ has included ancillary diagnostic tests.

123I-Meta-iodo-benzylguanidine scintigraphy ^123^I-MIBG) is a method that provides a functional analysis of the sympathetic postganglionic pathway, evaluating *in vivo* the cardiac noradrenergic neurotransmission. ^123^I-MIBG scintigraphy documenting cardiac sympathetic denervation is a supportive criterion for PD diagnosis, having over 80% specificity to distinguish PD from other parkinsonian conditions^21^. A study by Leite et al. showed that this method identified cardiac sympathetic neurotransmission impairment in Brazilian de novo PD patients without clinically defined dysautonomia^50^. The DLB consortium criteria also classifies ^123^I-MIBG as an indicative biomarker, showing good sensitivity (69%) and specificity (87%) values for discriminating probable DLB from probable AD^51^.

**Figure 2:**
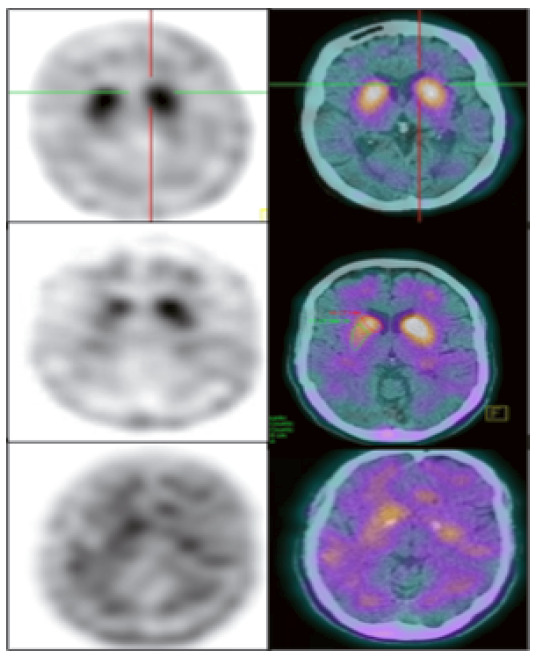
Dopamine transporter uptake in basal ganglia shown by SPECT or PET. Dopamine transporter uptake in basal ganglia demonstrated by TRODAT-1 SPECT imaging. The superior row shows a normal uptake in the caudate and putamen. The medium row shows an asymmetric uptake in a PD patient. The lower row shows a bilateral minimal uptake in a DLB patient. (Reproduced with permission from Dr. Artur Coutinho, Nuclear Medicine Center - Inrad - HC-FMUSP).

Moreover, normal functional neuroimaging of the presynaptic dopaminergic system is currently considered an absolute exclusion criterion for the MDS PD criteria. However, the task force mentioned that PD diagnosis does not require dopaminergic functional imaging^21^. Reduced dopamine transporter uptake in basal ganglia demonstrated by Single Photon Emission Computed Tomography (SPECT) or Positron Emission Tomography (PET) imaging is another indicative biomarker for DLB diagnosis. Dopamine Transporter (DAT) imaging has well-established utility in distinguishing DLB from AD, with 78% of sensitivity and 90% of specificity^52^. DAT imaging does not distinguish, however, between degenerative parkinsonian syndromes, such as progressive supranuclear palsy, corticobasal syndrome, frontotemporal dementia with parkinsonism, among others. Figure 2 shows TRODAT-SPECT images in PD patients.

PSG is the third indicative biomarker for DLB diagnosis. It aims to confirm the clinical suspicion of RBD, showing the loss of atony in the REM sleep stage^2^. A study by Sobreira et al. examined the association between cognitive status and presence of sleep disorders in PD patients by PSG, finding a significant association between global cognitive performance and wakefulness after sleep onset and the number of sleep stage changes^53^.

**Figure 3:**
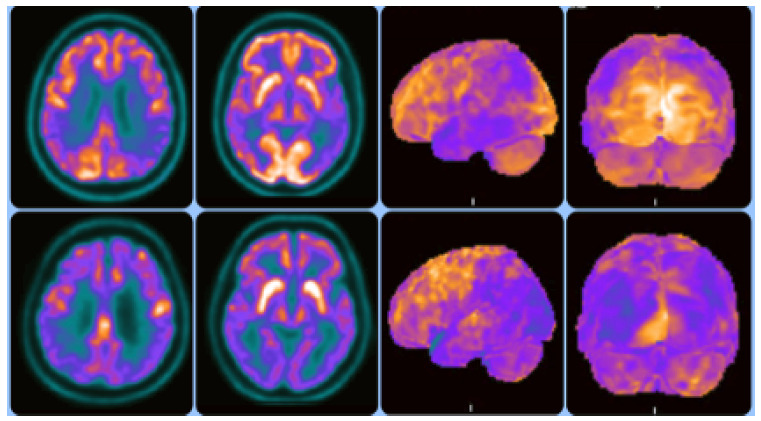
[^18^F]FDG-PET images in Alzheimer’s disease (AD) and Dementia with Lewy Bodies (DLB). Upper row - Images on the left with standard axial view and images on the right with [^18^F]FDG-PET 3D-stereotactic surface projection (3D-SSP, Cortex ID Suite software, GE Healthcare):occipital lobe metabolism is typically preserved in AD. Hypometabolism in AD usually occurs in bilateral temporoparietal regions. Lower row - Images on the left with standard axial view and images on the right with [^18^F]FDG-PET 3D-stereotactic surface projection (3D-SSP, Cortex ID Suite software, GE Healthcare): DLB shows occipital hypometabolism. Relative preservation of the posterior cingulate region, representing the “cingulate island sign”. (Reproduced with permission from Dr. Artur Coutinho, Nuclear Medicine Center - Inrad - HC-FMUSP).

Supportive biomarkers for DLB include: the relative preservation of medial temporal lobe structures on CT/MRI scans since patients with AD show greater atrophy of medial temporal lobe structures; the generalized low uptake on SPECT/PET perfusion/metabolism scan, which shows reduced occipital activity; and the posterior cingulate island sign on FDG-PET imaging. Figure 3 shows FDG-PET images in a DLB patient^54^. MCI in Parkinson’s Disease presents a similar typical pattern of hypometabolism, mainly in the posterior regions of the brain. Therefore, the absence of this typical pattern hints at an alternative diagnosis, including depression or an atypical parkinsonian syndrome^55^.

Furthermore, a prominent posterior slow-wave EEG activity with periodic fluctuations in the pre-alpha/theta range is a supportive biomarker for DLB diagnosis^56^. Some Brazilian studies have investigated the quantitative EEG changes concerning cognitive decline in PD patients, showing increased posterior theta and delta amplitude in patients with MCI-PD or PDD^57^ ^),(^ ^58^ and differences in EEG power and coherence in AD and PDD^59^ ^),(^ ^60^.

### Recommendation for primary and secondary care 

**Figure 4:**
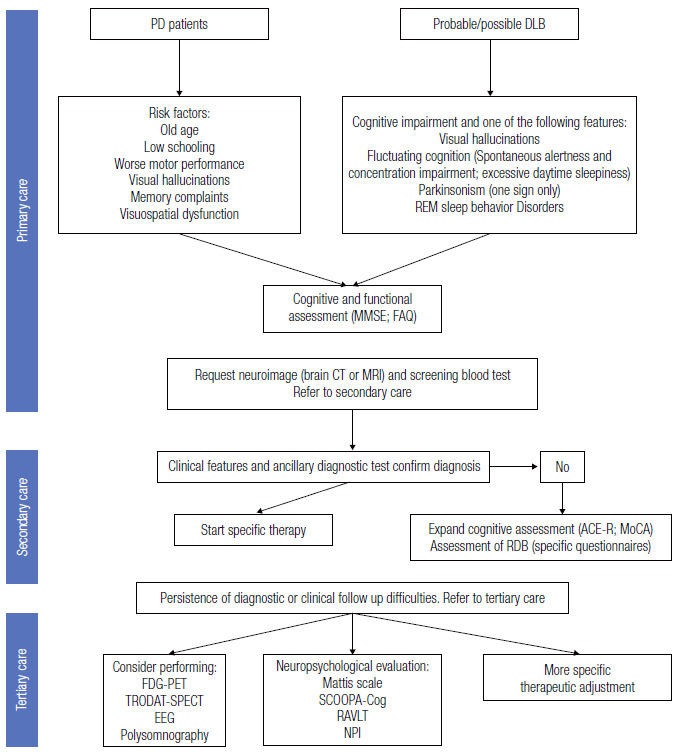
Flowchart of the approach to patients with parkinsonism and dementia.

Figure 4 presents a flowchart of the approach to patients with parkinsonism and dementia.
